# MiRNAs: A Powerful Tool in Deciphering Gynecological Malignancies

**DOI:** 10.3389/fonc.2020.591181

**Published:** 2020-10-23

**Authors:** Florentina Duică, Carmen Elena Condrat, Cezara Alina Dănila, Andreea Elena Boboc, Mihaela Raluca Radu, Junjie Xiao, Xinli Li, Sanda Maria Creţoiu, Nicolae Suciu, Dragoş Creţoiu, Dragoş-Valentin Predescu

**Affiliations:** ^1^ Fetal Medicine Excellence Research Center, Alessandrescu-Rusescu National Institute for Mother and Child Health, Bucharest, Romania; ^2^ Institute of Cardiovascular Sciences, Shanghai University, Shanghai, China; ^3^ Department of Cardiology, Jiangsu Province Hospital and Nanjing Medical University First Affiliated Hospital, Nanjing, China; ^4^ Cellular and Molecular Biology and Histology Department, Carol Davila University of Medicine and Pharmacy, Bucharest, Romania; ^5^ Department of Obstetrics and Gynecology, Polizu Clinical Hospital, Alessandrescu-Rusescu National Institute for Mother and Child Health, Bucharest, Romania; ^6^ Obstetrics, Gynecology and Neonatology Department, Carol Davila University of Medicine and Pharmacy, Bucharest, Romania; ^7^ Department of General Surgery, Sf. Maria Clinical Hospital, Carol Davila University of Medicine and Pharmacy, Bucharest, Romania

**Keywords:** miRNA, gynecology, cancer, signaling molecules, biomarkers, resveratrol

## Abstract

Accumulated evidence on the clinical roles of microRNAs (miRNAs) in cancer prevention and control has revealed the emergence of new genetic techniques that have improved the understanding of the mechanisms essential for pathology induction and progression. Comprehension of the modifications and individual differences of miRNAs and their interactions in the pathogenesis of gynecological malignancies, together with an understanding of the phenotypic variations have considerably improved the management of the diagnosis and personalized treatment for different forms of cancer. In recent years, miRNAs have emerged as signaling molecules in biological pathways involved in different categories of cancer and it has been demonstrated that these molecules could regulate cancer-relevant processes, our focus being on malignancies of the gynecologic tract. The aim of this paper is to summarize novel research ﬁndings in the literature regarding the parts that miRNAs play in cancer-relevant processes, speciﬁcally regarding gynecological malignancy, while emphasizing their pivotal role in the disruption of cancer-related signaling pathways.

## Introduction 

Short nucleotide RNA molecules with a length of 18 to 30 nucleotides, or microRNAs, have first been reported in 1993 (1–4) in studies based on genetic screening in nematodes. These findings have inspired researchers in biomedical sciences to focus on investigating these molecules in various organisms, focusing on their structure and functions. By now, it is well known that approximately 2% of the human genome comprises nearly 20.000–25.000 protein-coding genes, with most of the genome being composed of regions that do not encode but are transcribed into regulatory RNAs (ncRNAs), introns, and other sequences (4). These RNA sequences bear many functions, including the gene expression regulation of protein-coding genes at transcriptional level through transcript degradation, and at post-transcriptional level by translation suppression (5).

**Figure 1:**
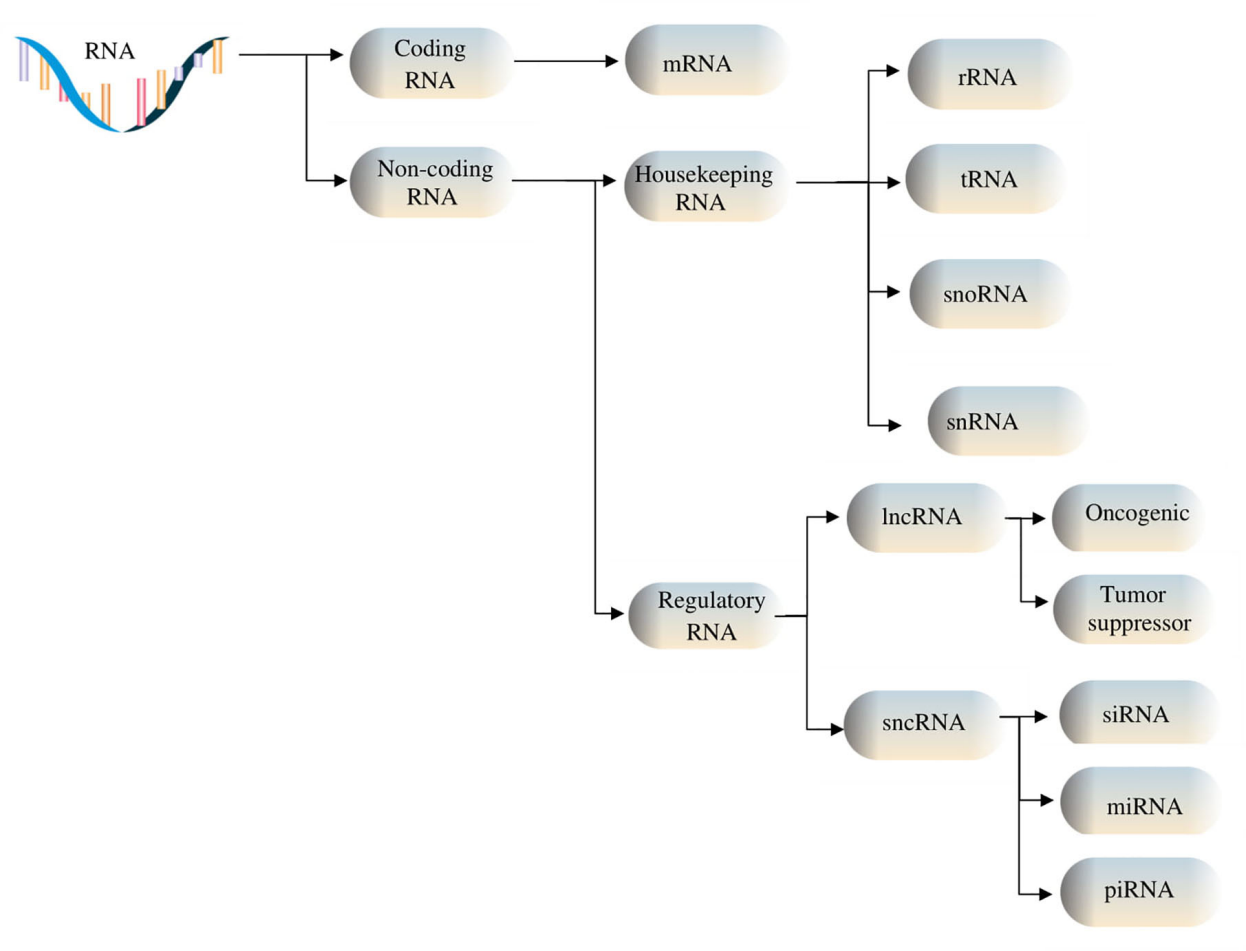
RNA types include coding and non-coding RNAs. Housekeeping ncRNAs are made up of ribosomal (rRNA), transfer (tRNA), small nucleolar RNAs (snoRNAs), and small nuclear (snRNA). Regulatory noncoding RNAs include short ncRNAs (sncRNAs) and long ncRNAs (lncRNAs), the former consisting of microRNAs (miRNAs), small interfering RNAs (siRNAs), and Piwi-associated RNAs (piRNAs), and the latter containing oncogenic and tumor suppressor lncRNAs.

Among non-coding RNAs (ncRNAs), there are two groups of RNAs fulfilling different tasks: some are indispensable for protein synthesis, such as ribosomal RNAs (rRNAs), transfer RNAs (tRNAs), small nuclear RNAs (snRNAs), and small nucleolar RNAs (snoRNAs), while others are involved in regulatory functions, for instance, Piwi-interacting ncRNAs (piRNAs), small interfering RNAs (siRNAs), microRNAs (miRNAs) and long non-coding RNAs (lncRNAs) (6, 7). The two classes of ncRNAs interact both with cellular components, in order to regulate various cellular processes and functions by controlling gene expression, and with each other, thus being consistently co-regulated (8). Depending on the number of specific nucleotides that each class of ncRNAs contains, ncRNAs are divided into lncRNAs, comprised of over 200 nucleotides, and sncRNAs, which are made up of less than 200 nucleotides. Furthermore, based on their functions, they can be divided into housekeeping, which are constitutively expressed RNAs, and regulatory RNAs, which are expressed during specific cell differentiation phases or as an answer to various modifications in the surrounding area (9), as depicted in **Figure 1**. The development of biomolecular techniques and big data analysis has allowed the identification of the functions that these types of RNA fulfill, as well as their interaction with various subcellular components, and their regulation. For instance, lncRNAs interact with miRNAs at specific binding sites, resulting in their mutual regulation and messenger RNA (mRNA) transcript control (10). Furthermore, it has been shown that ncRNAs form interconnected networks that regulate numerous physiological and pathological biological processes, including protein synthesis, gene regulation, chromatin modulation, tumor cell invasion – with multiple studies currently focusing on their roles as either oncogenic or tumor suppressor factors (11–13). These networks also govern the expression of snoRNAs, which can act as precursors for other RNA types such as piRNAs (14).

snoRNAs often originate within the nucleolus, measuring approximately 60–300 nucleotides (nt) in length. snoRNAs are generally encoded within the intronic regions as long non-coding RNAs or can be separately transcribed from the intergenic regions (13). Overall, snoRNAs can be classified into two main groups, namely C/D box snoRNAs (SNORDs), and H/ACA box snoRNAs (SNORAs) (15). snoRNAs contribute to the biogenesis and maturation of rRNA, as well as in the complex interaction between snRNAs, tRNAs, and rRNAs. Individually, SNORAs participate not only in the pseudouridylation process by linkage with SNORDs and the dyskerin nuclear protein (15), but also in the methylation cycle along with fibrillarin proteins (16). However, some snoRNAs lack defined functions, being generally referred to as orphan snoRNAs (17, 18).

While miRNAs are currently considered as the most plentiful circulating ncRNAs in normal and cancer patients, data from an RNA-seq analysis profiling extracellular RNA from both cancer patients and healthy controls has shown that piRNAs amount to almost the same sum (19). Though it has been shown that the synthesis of piRNA does not require the aid of the Dicer enzyme (20), their exact roles are yet to be fully elucidated (21). However, the interaction between miRNAs and piRNAs is known to result in the regulation of gene expression by targeting mRNAs (8).

miRNAs are small non-coding RNA molecules that function as post-transcriptional regulators of gene expressions (5, 22, 23). They are highly important in both physiological and pathological processes in the human body, as well as in cell development and proliferation, tissue differentiation, and programmed cell death (24), while also being involved in maintaining 60 to 70% of the cellular homeostasis (7). miRNAs are abnormally expressed in a multitude of diseases, such as cardiovascular and renal illnesses (25, 26), muscle disorders (27), some types of fibrosis (28, 29), pre-diabetes and diabetes (30, 31), leukemias and hematological malignancies, as well as disorders involving hematopoietic stem cells and stem cell differentiation (32–36). Furthermore, miRNAs have also been revealed to facilitate the maintenance of the blood-brain barrier, thereby mediating central nervous system homeostasis (37–39). Last but perhaps foremost, miRNAs have been extensively studied in a wide variety of malignancies (40, 41). To date, approximately 500–1,000 different mammalian miRNA genes are known (25). A complete list with specific details regarding the nomenclature and annotation of miRNA sequences was founded in the year 2002, later known as the microRNA Registry. Nowadays, the miRbase online instrument can be used, providing information about microRNA sequences from 271 organisms, with 38.589 hairpin precursors and approximately 48.860 mature microRNAs (4, 42).

Cancer cells differ from normal cells mainly due to their ability to divide and grow uncontrollably, as a result of modifications undergone by specific genes. Considerable gene variation and altered pathways have been reported for different types of cancers, which depend on the genetic individuality of the affected organism as well as on epigenetic factors (4). Understanding the mechanisms and signaling pathways by which genes become mutated is therefore essential in order to enhance the chances of establishing personalized therapeutic schemes. To this extent, in the last years, miRNAs have emerged as signaling molecules in a multitude of biological signaling pathways in distinct types of neoplasia, and it has been demonstrated that they can regulate cancer-relevant processes. Due to the capacity of a single miRNA molecule to target hundreds of mRNAs, aberrant miRNA expression can be held responsible for the dysregulation of at least several cancer-related signaling pathways (41).

Gynecological cancers pose an important public health issue, being some of the most frequent cancers among women of all ages (43). Patients are oftentimes diagnosed in late stages not only due to a general lack of awareness and knowledge about cancer but sometimes also because of improper screening and even misdiagnoses (44). In gynecological cancers, several signaling pathways have been identified to be modified, including the transforming growth factor-β (TGF-β)/Smad pathway, G Protein-Coupled Receptors (GPCRs), phosphatidylinositol-3-kinase (PI3K)/Akt, the mechanistic target of rapamycin (mTOR), the mitogen-activated protein kinases (MAPK) and the extracellular signal-regulated kinases (Erk), fibroblast growth factor (FGF), the insulin receptor (IR) and insulin-like growth factor receptor (IGFR), vascular endothelial growth factor (VEGF), Toll-like receptors (TLRs), Wnt/β-catenin, Jak/STAT, the Notch signaling pathway, the nuclear factor kappa B (NF-κB) pathway. Other signaling pathways that are implicated in several pathologies including breast cancer, are related to the nuclear receptor superfamily of ligand-dependent factors such as the estrogen receptor (ER), the retinoic acid-related orphan receptors (ROR α-γ or NR1F1-3), and the orphan receptor TAK1 (TR4 or NR2C2) (45).

In this review, we have summarized the implications and future perspectives regarding the signaling functions of miRNA and their roles in regulating oncogenic processes in breast, ovarian, cervical, vulvar, and endometrial cancer.

## miRNA Biogenesis

microRNAs have long been shown to control numerous biological processes, including tumorigenesis, with miRNAs being massively dysregulated in tumor cells (46). While the dysregulation of miRNAs is well documented in a range of diseases, direct causal links in cancer have relatively recently been elucidated. Tumoral cells often associate reduced levels of mature miRNAs as a consequence of genetic loss and epigenetic gene silencing resulting in defects in their synthesis (47).

**Figure 2:**
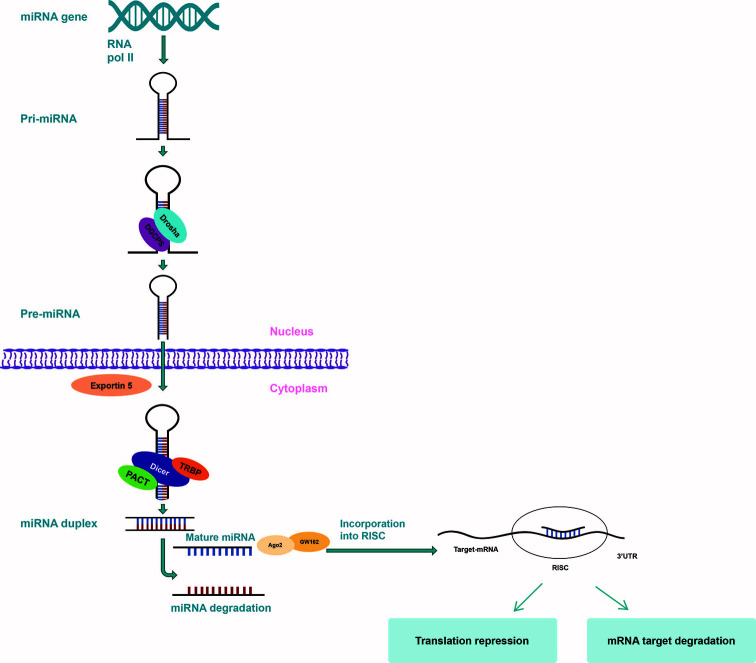
miRNA biogenesis. miRNA gene is transcribed by RNA polymerase II to form a hairpin loop primary transcript (pri-miRNA) which is processed by Drosha/DCGR8 to form pre-miRNA. pre-miRNA is then exported to the cytoplasm by exportin 5, where Dicer cuts off the hairpin loop so as to create a complex that includes the mature miRNA. The mature miRNA is next incorporated into RISC to target the 3’-UTR site of the mRNA to silence expression by cleavage or regression.

The biogenesis of microRNAs results from a well-defined conserved processing mechanism, with deviations being associated with several diseases (48, 49). Following experiments on mice, primary miRNAs (pri-miRNAs) represent the basis of creation for miRNAs, which is a process that takes place in two phases, the first one taking place in the nucleus and the second one in the cytoplasm, both being governed by the specialized RNase type III proteins, Drosha and Dicer (**Figure 2**) (50). The fundamental RNA polymerase that is responsible for the transcription of miRNA genes is RNA polymerase II (Pol II). Pol II-dependent miRNA gene expression has a short-term control, in order to enable the synthesis of a specific group of miRNAs per certain conditions and cell types (51–53). pri-miRNA transcripts contain one or more local hairpins that are cleaved by the nuclear RNase III enzyme Drosha and its partner, the DiGeorge syndrome critical region 8 (DGCR8) protein (54, 55) in pre-miRNA sequences made up of almost 80 to 100 nucleotides (56, 57). This step of miRNA biogenesis pathway is localized in the nucleus and requires DGCR8 in order for a large complex, known as the Microprocessor complex, to be formed (56). Following transcription, the pre-miRNA is displaced from the nucleus towards the cytoplasm by Exportin-5 (58, 59). In the cytoplasm, Dicer, a cytoplasmic ribonuclease, cleaves the pre-miRNA into a double incomplete mature miRNA (a miRNA/miRNA duplex made up of approximately 20 to 22 nucleotides) (24, 60).

Within the cytoplasm, incomplete miRNAs suffer additional modifications in order to become mature miRNAs, being processed by Dicer and RNase III type protein and loaded onto the Argonaute (Ago) protein so as to generate the effector RNA-induced silencing complex (RISC). The mature miRNA duplex is included in RISC, which further coordinates the translation of complementary mRNA and guides it to target miRNA (53, 61). The mature miRNA identifies its correspondent sequences in the 3′ untranslated region (3’-UTR) of their target mRNAs by way of seed region, typically placing nucleotides 2–7 in the miRNA (62, 63). One strand of the produced RNA duplex is subsequently loaded to RISC while the other strand is typically degraded. In some cases, some pre-miRNAs produce mature sequences from both strands that survive and are functional in comparable frequencies (64). Since regulation does not require high complementarity, only one miRNA can target up to hundreds of different mRNAs, leading to the development of aberrant miRNA expression, affecting a multitude of transcripts that have great repercussions on cancer-related signaling pathways. Additionally, miRNAs can trigger downstream signaling pathways by directly binding with Toll-like receptors (TLRs) working as ligands (65–67).

## miRNA and Signaling Pathways in Cancer

In just a few years, microRNAs have become strongly fixed as key molecular components of the cell in both pathological and normal states (68). The main activity of miRNAs is to lead protein translation by linking to complementary sequences of the 3’-UTR sites of target mRNAs and by negatively regulating mRNA translation (69). The first proof of miRNA involvement in human malignancies was provided by Croce’s research group, which aimed to find tumor repressors at chromosome 13q14 site in B-cell chronic lymphocytic leukemia cells (70). This site carries miR-15a and miR-16-1 genes and it has been found to be frequently deleted or downregulated in B-cell chronic lymphocytic leukemia. Both miR-16-1 and miR-15a serve as tumor repressors that promote cell death by suppressing B-cell lymphoma 2 (Bcl-2), an anti-apoptotic protein heavily expressed in malignant non-dividing B cells and other solid malignancies (71, 72).

An abundance of scientific research has lately been published, concerning the function of miRNAs in gynecological malignancies. For instance, miRNAs such as miR-145 have been identified as central players in cervical carcinogenesis, whereas it has been demonstrated that miR-125b, miR-145, miR-21, and miR-155 have pivotal roles in breast malignancies (73). miR-200 and let-7 have been identified as key modulators in ovarian neoplasms, while miR-185, miR-210, miR-423, let-7c, miR-205, and miR-429 have been associated with oncogenesis, invasion, and metastasis in endometrial carcinomas (74, 75). miRNAs target cell-cycle elements and control various signaling pathways in several physiological and pathological processes, including gynecological malignancies, thus being involved in cell proliferation. Signaling pathways in which miRNAs have been shown to be involved and their target genes have been summarized in **Table 1**.

**Table 1:** miRNA signaling pathways involved in gynecological cancers.

miRNA	Signaling pathway	Target	Target expression	Action	Pathology	Reference
miR-433	MAPK	RAP1A	Overexpression	Cell migration, proliferation, apoptosis	Breast cancer	(76)
miR-99a	mTOR FGFR3	PI3-AKT	Overexpression	Invasion, proliferation, apoptosis	Cervical cancer Breast cancer	(77)
miR-155	AKT	LKB1	Overexpression	Autophagy	Cervical cancer	(78)
miR-21	TNFR1 PI3K/AKT/mTOR RAS/MEK/ERK	Caspase 3 TNF-alpha PTEN RASA1	Overexpression	Apoptosis	Breast cancer Cervical cancer Ovarian cancer	(79)
miR-200 miR-141 miR-200a miR-200b miR-200c	NOTCH TGF-beta	ZEB1 ZEB2 E cadherin EMT	Overexpression	Invasion, metastasis	Ovarian cancer	(80)
Let-7 *miR let-7d-5p	RAS HGMA1	P53	Overexpression	Apoptosis	Ovarian cancer	(81)
miR-34a	p53	HNRNPA1		Cell proliferation	Breast cancer Endometrial cancer	(82)
miR-424	p53	HNRNPA1	Overexpression	Cell proliferation, apoptosis	Breast cancer	(82)
miR-503	p53	HNRNPA1	Overexpression	Cell proliferation, apoptosis	Breast cancer	(82)
miR-142-3p	Bach-1	EMT	Overexpression	Invasion, migration	Breast cancer	(83)
miR-205	ZEB1, ZEB2	EMT PTEN	Overexpression	Apoptosis, cell differentiation, and proliferation	Endometrial cancer	(84)
miR 4712-5p	PTEn/AKT/GSK3beta/cyclin D1	PTEN	Overexpression	Cell invasion, metastasis	Vulvar cancer	(85)
miR-3147	TGF-β/Smad	TGFβ RII EMT	Overexpression	Invasion, cell proliferation, migration	Vulvar cancer	(86)
miR-146a		BRCA1	Overexpression	Cell proliferation	Breast cancer	(87)

ZEB1 and ZEB2 Zinc finger E-box-binding homeobox 1/2 HNRNPA1 Heterogeneous nuclear ribonucleoprotein A1.

Toll-like Receptors (TLRs),;, are membrane-bound receptors found on antigen-presenting cells (APCs) and they are members of the group of pattern recognition receptors (PRRs). Some signaling pathways, such as the interferon regulatory factor (IRF), ERKs, NF-κB, MAPKs, c-Jun N-terminal kinases (JNKs), p38, are activated following TLRs stimulation, being involved in the immune response (88).

Transforming growth factor-beta (TGFβ) is part of a large family of growth and differentiation factors that perform multiple functions in embryonic development or act as cytokines in the postnatal period, being divided into two functional groups: TGFβ and the growth/differentiation factor (GDF) group. The key target genes of the TGF-β signaling pathway are the receptor-regulated SMADs (89, 90). In vulvar carcinoma, Yang X et al. have linked the overexpression of miR-590-5p with the downregulation of the target gene TGFβIIR, which induced the appearance of malignant cellular changes and metastasis in sentinel lymph nodes. TGF-β signaling is also involved in other cancers, including breast and endometrial neoplasms (45, 89, 91).

GPCRs contain seven transmembrane regions, making up the largest signaling receptor family. They exert their actions by activating the phosphatidylinositol bisphosphate (PIP2) and cAMP signaling pathways (92). These signaling pathways are involved in different physiological and pathological functions, such as cell proliferation and invasion, being described in numerous cancers, including ovarian and breast cancers (45, 93).

The PI3K/AKT pathway further plays an important role in the survival of tumor cells, metabolism, and growth regulation, with some of the most common mutations in cancer being associated with deviations of this signaling pathway. Its disruption affects both the EGFR/HER family and the mTOR pathway. This signaling pathway is frequently altered in ovarian, cervical, endometrial, and breast cancer (45, 94, 95). For instance, in cervical cancer, miR-21 can increase cell growth via the PI3K/AKT/mTOR signaling pathway, by binding and inhibiting the tumor suppressor PTEN (96). Further on, miR-486, which is substantially downregulated in non-small cell lung cancer, has been demonstrated to alter migration and proliferation via the IGF-1/PI3K/Akt pathway, by targeting IGF1, IGR1R, and p85 (97, 98).

miR-21, on the other hand, promotes cell proliferation, through the Ras/MEK/ERK signaling pathway, which is inhibited by miR-21 targeting the 3’-UTR of RASA1 mRNA in ovarian cancer (99, 100). The MAPK and ERK molecules operate in a signaling cascade defined as the MAPK cascade. MAPK/ERK pathway is downstream of some transmembrane receptors, such as EGFR, FGFR, VEGFR, IGFR, and GPCR, and it is involved in essential functions like development, proliferation, apoptosis, or differentiation of cells in ovarian and endometrial cancer (45, 73, 101).

The Notch signaling pathway is essential in cellular processes and it is activated in response to cell-cell contacts. Notch receptors are transmembrane proteins consisting of a series of different protein molecules. Notch activation is involved in the regulation of gene expression that is implicated in survival, proliferation, and differentiation of cells (102). Through genome-scale sequencing, recent studies have revealed that mutations in the Notch genes could be identified in a broad spectrum of cancers. They further found that resveratrol has an inhibiting action when Notch signaling is oncogenic, while it increases the tumor-suppressive effect when Notch signaling has suppressive tumoral action (45, 103).

CTCF (CCCTC-binding factor) is a zinc-finger protein gene capable of targeting numerous binding sites within the genome, acting both as a transcriptional activator and repressor (104). Furthermore, it can also serve as an insulator, impeding the communication between promoters and enhancers (105). In this manner, and due to its ability to establish inter- and intrachromosomal bonds, CTCF can either up- or down-regulate the expression of a substantial number of target genes, depending on the context, thus fulfilling diverse roles in epigenetic modulation (106). Moreover, when cooperating with chromatin architectural proteins such as cohesin, the resulting complex governs the spatial organization of the genome (107). In addition to these roles, there is an increasing body of evidence suggesting the involvement of CTCF in the regulation of certain miRNAs (106, 108, 109). Specifically, by binding to the CpG sites of miR-375, CTCF manages to silence its expression in estrogen receptor (ER) negative breast cancer cells. As miR-375 is a key driver of cell proliferation, these findings confirm the tumor suppressor role of CTCF in breast cancer (106, 108, 110). Furthermore, silencing of tumor suppressor miR-125b1 in breast cancer due to epigenetic phenomena that result in the methylation of CpG islands preventing CTCF binding, leads to aberrant cell proliferation (108, 109, 111).

### miRNAs, BRCA Mutations, and Breast Cancer

Breast cancer is the most common malignancy among women worldwide, with 5 to 10% of patients carrying an inherited predisposition (112). The breast cancer 1 and 2 (BRCA1/2) genes are tumor suppressor genes responsible for the synthesis of proteins involved in damaged DNA repair (113). Mutations in either gene have been associated with significantly increased risk of both breast and ovarian cancer (114), and, to a lesser degree, other types of cancer, including prostate and pancreatic cancer, especially in BRCA2 mutations (115). Specifically, women with germline BRCA1/2 mutations face risks of up to 72 and 69% respectively of having breast cancer by the age of 80 (116, 117). While numerous BRCA genes variants are possible, not all of them carry the same risk, with studies establishing four main pathogenic mutations: single nucleotide mutations (SNPs) resulting in premature termination codons (PTCs) (118), large in-frame deletions or insertions of ≥ 1 exon, transcription regulatory region deletions (114) and certain pathogenic missense variants (119, 120). However, recent whole genome association studies (WGAS) using targeted RNA sequencing have enabled the analysis of multi-exonic ncRNAs in breast cancer samples and, although their functionality has not been revealed yet, they constitute promising leads in better grasping the etiology of breast cancer (121).

The expression of up to thirty miRNA has emerged as having direct consequences on all phases of breast cancer, from formation to progression and propagation (122, 123). Together with the group of miRNAs acting as tumor suppressors that delay or block the potential to cause cancer, there are other oncogene miRNAs (onco-miRNAs) that can cause neoplastic transformation when overexpressed. Breast cancer can be caused by genomic instability as a result of alterations that accumulate in the human genome. One of the processes that can lead to genome instability causing DNA damage is represented by defects in the repair pathway such as double-strand brakes (DSBs), which tend towards cell apoptosis. Considering the crucial role of BRCA1/2 in breast cancer suppression, due to their function in maintaining genome integrity through protein synthesis required for repairing DNA damage, both BRCA genes play parts in apoptosis and the processes of tumor suppression (124, 125). Over 100 miRNAs target the transcription of messenger RNA from the BRCA1 gene. Chang and Sharan have proven that seven miRNAs target BRCA1 (126). miR-146-5p and miR-146a deleting BRCA1 may cause the development of sporadic basal-like and triple-negative breast cancer (127, 128). Negative feedback between BRCA1 miRNA and miR-146a has been described, in which the BRCA1 translation is inhibited by miR-146a and, in turn, miR-146a is up-regulated by BRCA1 (127). Another well-known onco-miRNA is miR-155, which is involved in breast tumor formation and spreading, found more often in inflammation-based cancers and neoplastic transformation caused by inflammation (129). The pathogenesis of breast cancer is influenced by the DNA methylation of miRNA genes, with BRCA1 functionality reduction inducing global hypomethylation. These findings highlight possible treatments of BRCA1-deficient breast tumors that may be developed by targeting miR-155, due to its impact on BRCA1 mutation carriers (126, 130–132).

Several proteins acting as BRCA1/BRCA2 stability regulators have been identified, including the cysteine protease Cathepsin S (CTSS), which reacts on BRCA1 with BRCT domain, initiating the process of proteolytic degradation (133), E3 ubiquitin-protein ligase HERC2, F box protein 44 (FBXO44) and E2 ubiquitin-conjugating enzyme E2T (UBE2T). FBXO44 ubiquitination downregulates the BRCA1 protein (134, 135). BRCA1 expression stabilization is gained through interaction with BARD1 protein and the reduction of proteasome sensitive ubiquitination. The BRCA1 protein level is increased as the proteasomal degradation is prevented by IGF-1 receptor signaling due to AKT-dependent phosphorylation of BRCA1 in response to estrogen. Recent research performed by Kim et al. has shown that BRCA1 is directly phosphorylated by the Fyn related kinase (Frk/Rak) and, as a result, BRCA1 protein stability is positively regulated (133). Besides protein level regulation, BRCA1 and BRCA2 have been found to participate in a complex regulatory post-transcriptional program. For instance, miR-19a and miR-19b interact with 3’-UTR of BRCA2 mRNA resulting in a simultaneous decrease of protein levels and mRNA of BRCA2. In chronic myeloid leukemia cells, expression of BCR-ABL1 oncoprotein is linked with BRCA1 downregulation. Recent studies revealed that the TIA1 cytotoxic granule-associated RNA-binding protein-like 1 (TIRA) is responsible for BRCA1 downregulation, which disables mRNA translation of BRCA1 by linking to adenylate-uridylate-rich elements (ARE) sites in the 3’-UTR of BRCA1 mRNA. The study also described the complex formed between TIRA, the mRNA binding protein Hu antigen R (HuR), and BRCA1 mRNA (136).

Further on, more recent studies performed by Gorrini and colleagues have demonstrated that BRCA1 deficient cells are protected by estrogen from reactive oxygen species induced death through the activation of PI3K/AKT and NRF2 upregulation (nuclear factor erythroid 2 related factor 2) transcriptional program. In consequence, antioxidant genes are increased (137–139). The results showed that a local upregulated estrogen concentration helps the expansion and survival of BRCA1 mutated breast cancer cells (140). Furthermore, hormone functions are affected by BRCA1 in different ways such as activating Era expression (141), adjusting the level of progesterone receptor (PR) (142, 143), and repression of estrogen-dependent gene transcription (144, 145).

In breast cancer, studies have shown that various signaling pathways are implicated in the proliferation as well as cellular death of malignant cells. For instance, Zhu et al. have shown that one significant role in breast cancer growth is played by the signaling pathway of mTOR (mammalian target of rapamycin) (146), its downregulation by miR-100 and/or miR-125b enabling cellular death and inhibiting the progression of breast cancer (147, 148). Another miRNA, miR-142-3p, belonging to the miR-142 family, might be related to the development of various types of malignancies, especially breast cancer, by targeting various mRNAs, including Bach1, which is highly active in cancerous cells. To this extent, in their study, Liang et al. have found that increased mRNA levels of Bach1 were considerably linked to poor metastasis-free-survival rates (149). Further studies have also indicated that overexpression of miR-142-3p in breast malignancies resulted in the downregulation of Bach-1, making it likely that miR-142-3p could be a target in breast cancer therapy (83, 149, 150).

In previous studies, miR-433 has been found to have acted as an oncogene - for instance, in colorectal cancer, overexpression of miR-433 downregulates MACC1 and leads to cell death, while in hepatocellular carcinoma, it suppresses cell proliferation by targeting HDAC6, PAK4, and GRB2 (151–153). In breast cancer, miR-433 has generally been found to be decreased, while its overexpression has been linked to cell death and inhibition of tumor cell growth and migration. After screening miRNA target genes, T. Zhang et al. predicted Rap1a as a potential target of miR-433, later proceeding with their experiment. Consequently, they found that cells transfected with miR-433 associated decreased Rap1a protein levels along with slightly lowered Rap1a mRNA levels, thus demonstrating that, by targeting the RAP1A gene and subsequently activating the MAPK signaling pathway, miR-433 behaves as a tumor suppressor (76). The MAPK pathway is known to be implicated in tumorigenesis, playing key roles both in the growth and apoptosis of malignant cells (154), however, in breast cancer, the RAP1A/MAPK cascade remains to be further clarified (155).

Past studies have shown miR-99a to take part in the pathology of several cancers, such as non-small lung cell carcinoma, leukemia, and prostate cancer (156–158). Long et al. have later found that miR-99a also plays a significant role in breast neoplasia, where it acts as a regulator of fibroblast growth factor receptor 3 (FGFR3). They proved for the first time that miR-99a directly targeted FGFR3 in breast malignancies and that it could be used as a convenient biomarker for this pathology (77). FGFR3 is also upregulated in various types of tumors, and its abnormal expression could initiate distinct signaling pathways, like the PI3-AKT and the FGFR3 signaling pathways, this way contributing to the development of cancer (159, 160). Several studies have also shown that miR-99a is downregulated in malignant tumors like esophageal carcinoma, head and neck squamous cell carcinoma, cholangiocarcinoma, and also in primary breast tumors compared to normal breast tissue (77). On the other hand, researchers demonstrated that the upregulation of miR-99a in breast cancer inhibits malignant cell proliferation and invasion (77, 161–163), miR-99a working as a tumor suppressor. Further studies are therefore desired before potentially implementing miR-99a as a diagnostic and prognostic biomarker.

In breast cancer, snoRNAs have also been highlighted as having prognostic applicability, including SNORD89 and SNORD46. In this regard, in their thorough NGS analysis, Krishnan et al. have recently found that SNORD89/46 were the most significantly downregulated snoRNAs in breast cancer patients, thus reporting them as prognostic markers for breast cancer (164). Further studies have reported small nucleolar RNA-derived RNA-93 (sdRNA-93), a processed stable form of snoRNA-93, as playing an important role in cell invasiveness in epithelial human breast cancer cell lines. At the same time, sdRNA-93 was significantly higher expressed in Luminal B/HER2+ breast cancer samples when compared to normal breast tissue and other types of breast cancer (165, 166).

Further on, it has been discovered that piR-36712 plays a pivotal role in suppressing breast cancer cell proliferation through the retroprocessed pseudogene of selenoprotein W (SEPW1), SEPW1P, by inhibiting the expression of SEPW1. The expression of both p21 and p53 was inhibited by the mRNA degradation induced by SEPW1. Concurrently, piR-36712 has been found to promote the antineoplastic effect of chemotherapeutic agents (167). A recent study carried out by Fancello and colleagues has found that, in approximately 34% of invasive breast cancer samples, ribosomal alterations were driven by mutations in the uL18/RPL5 ribosomal protein genes, thus highlighting the suppressor role of RPL5 in breast cancer (168).

Clearly, ncRNAs remain a vast unexplored resource for the better understanding of breast cancer tumorigenesis and metastasis, potentially aiding in the progress of identifying relevant diagnostic and prognostic markers, as well as therapeutic targets.

### miRNAs in Ovarian Cancer

Ovarian cancer is the most lethal gynecologic neoplasia, associating a very poor life prognosis. Epithelial ovarian cancers amount to more than 90% of this malignancy, and the 5-year survival rate is just 29% (169). Moreover, ovarian cancer is diagnosed late due to the absence of noticeable symptoms in the early stages, and, when detected, carcinomatosis spread is higher than 60% (170, 171). Biomarkers used today for prediction and prognosis are CA-125 and human epididymis protein 4 (HE4), used along with imaging and screening methods. However, the relatively low sensitivity and specificity of these procedures require the discovery of new, more efficient diagnostic and prognostic methods. To this end, many studies have been performed and more are underway, in order to explore the exact relation between miRNAs and ovarian cancer and to improve current diagnosis, prognosis and treatment methods.

The development of tumors is imposed by the tumor microenvironment. Extracellular matrix molecules regulate cancer invasion and metastasis, and, at the same time, down-regulation of miRNAs controls the spread of the tumor by degrading the extracellular matrix (172, 173). Matrix metalloproteinases are important in tumor aggressiveness and increase cancer metastasis by causing deterioration in the molecules of the extracellular matrix (174). Studies have demonstrated that MMP-9, MMp-3, MMp-7, MMP-2 are involved in tumor aggression in ovarian cancer. An example is that of MMP-7, which is increased in malignant ovarian tissues, where miR-543 is substantially reduced. The explanation resides in the fact that miR-543 decreases MMP-7 transcription by attaching to the 3’-UTRs of MMP-7 mRNA, leading to the reduction of cancer proliferation (175).

The miR-200 group consisting of miR-141, miR-200a, miR-429, miR-200c, and miR-200b, is clustered in chromosome 1 (1p36) and adjusts many cellular functions, including cell death, proliferation, and epithelial-to-mesenchymal transition (EMT). This group decreases the transcriptional suppressors of E-cadherin, ZEB1, and ZEB2, promotes E-cadherin expression, and modulates the conversion of mesenchymal cells into epithelial cells (176, 177). miR-200a can bind to three specific sites within the 3’UTR region of the ZEB1 mRNA, while miR-141 has two potential binding sites in the 3’UTR region of the ZEB2 mRNA. By binding to the ZEB1 and/or ZEB2 mRNAs, these miRNAs mediate the post-transcriptional inhibition of the ZEB1 and ZEB2 gene expression (177, 178). Furthermore, negatively regulated miR-200a in cancer cells inhibits E-cadherin, known invasion, and metastasis suppressor (179). Tumor angiogenesis is another process influenced by the miR-200 family. Studies have shown that new blood vessel development is inhibited by this cluster’s influence on the interleukin-8 (IL8) secreted by cancer cells and on chemokine CXCL-1, thus decreasing the spread of tumor cells through blood circulation (180). To this extent, Pecot et al. have implemented several experimental models, demonstrating that tumor-targeted delivery of miRNAs of the miR-200 family leads to a significant decrease in angiogenesis and consecutive tumor cell metastasis, while also promoting vascular normalization (181).

The miRNA let-7 family is another important cluster which has been broadly studied and has a significant function in ovarian cancer growth. It has been demonstrated that, by decreasing the expression of specific proteins like c-Myc, Ras, cyclin 2, and the High Mobility Group AT-Hook 2 (HMGA2) protein, the let-7 group decreases cell proliferation and supports both apoptosis and cell differentiation in various types of cancer. Since the let-7 group is poorly expressed in aggressive ovarian malignancies, it can be concluded that it may decrease the infiltration and spreading of ovarian cancer (81). miR let-7d is known to behave as an oncogene in ovarian malignancies, and its inactivation can lead to the overexpression of Ras, resulting in apoptosis of cancer cells (182, 183). One study performed by Chen Y. et al. found that miR let-7d-5p negatively regulated HGMA1 in ovarian cancer, leading to the obstruction of the p53 signaling pathway, thus suppressing cell proliferation and facilitating programmed cell death (184). Furthermore, in wanting to predict chemotherapy resistance and prognosis of epithelial ovarian cancer, Xiao et al. have performed thorough research focusing on the human let-7 family. They found that let-7e inhibitor had an up-regulatory effect on the mRNAs of target genes regulatory factor X 6 (RFX6), enhancer of zeste 2 (EZH2), caspase 3 (CASP3), and matrix metalloproteinase-9 (MMP9). On the other hand, treatment with let-7e mimics resulted in decreased mRNA levels of poly-ADP-ribose-polymerase 1 (PARP1) and insulin-like growth factor-1 (IGF-1). Further on, they found that ovarian cancer cell lines had an increased sensitivity to cisplatin when associated with overexpression of let-7e, thus confirming the role of poor let-7e expression in platinum resistance in epithelial ovarian cancer (185).

### miRNAs in Cervical Cancer

Cervical malignancies pose serious health risks to the female population, associating a poor prognosis with an overall 5 years survival rate amounting to less than 40% (186), thus highlighting the need for new diagnostic methods and targeted treatments. Studies have shown that the abnormal expression of miRNAs contributes to the development of cervical cancer, due to their innate ability to regulate tumor promoter and/or repressors genes (187, 188). To this extent, although limited information regarding cervical tumors is currently available, miR-433 has been demonstrated to be downregulated in cervical tumoral tissues and cell lines, as opposed to normal tissues, its levels reflecting tumor characteristics such as size, stage, and dissemination (189, 190). While the upregulation of miR-433 in cervical cancer inhibits cell proliferation and invasion and promotes cell death, rescue experiments have demonstrated that metadherin (MTDH), an oncogene that facilitates cancer cell migration and metastasis, is a direct target gene of miR-433. In this regard, the overexpression of MTDH has been shown to reverse the effects of the overexpressed miR-433 in cervical cancer cell lines (191). Employing functional studies, Liang et al. have demonstrated that cervical cancer cell lines treated with miR-433 agomir substantially decreased mRNA levels of MTDH, thus inhibiting tumor cell proliferation and invasion and triggering apoptosis (192). MTDH has also been previously demonstrated to influence the regulation of β-catenin and AKT pathways and, due to its inhibitory effect on these pathways in cervical cancer, it has been validated as a direct target of miR-433 (193, 194).

Additional studies have revealed that miR-21 acts as an oncogene in various malignancies, by regulating several pathways involved in tumor progression (195). In cervical cancer, its overexpression acts as a gene expression inhibitor (196). miR-21 upregulates mRNA and protein expression levels of TNFα (197), which initiates cellular apoptosis by binding and activating the TNFR1 receptor in HeLa cells, thus inhibiting the TNFR1 pathway (198). In contrast, the TNFR2 pathway is increased by miR-21, and cell proliferation is activated by TNFα, which binds TNFR2, upregulating NF-κB, and thus inhibiting Caspase 3 and activating JNK (197). Further on, miR-21 has also been shown to increase cell proliferation via the PI3K/AKT/mTOR signaling pathway, by binding and inhibiting the tumor suppressor PTEN (96, 199). PTEN acts as a negative regulator of this particular signaling cascade, by targeting AKT and therefore modulating cell differentiation, proliferation, and migration (96). Loss of miR-21 leads to considerable upregulation in PTEN mRNA levels, with Chen et al. demonstrating that cells lacking miR-21 display a lesser degree of cisplatin resistance, both in culture and xenograft mouse models (200). Furthermore, miR-21 also promotes cell proliferation through the Ras/MEK/ERK signaling pathway, which is inhibited by miR-21 due to its targeting of the 3’-UTR of RASA1 mRNA (99, 100, 201). However, further investigation is required in order to elucidate other targets of miR-21, as well as their exact implications in cervical malignancy.

### miRNAs in Endometrial Cancer

In developed countries, endometrial cancer is the most frequently-occurring gynecological cancer, having to do with increased obesity rates, longer life expectancy, and particular lifestyles in these nations. Globally, it is the second most harmful type of cancer among women, after cervical cancer (202), with the most common type of endometrial cancer being the endometrioid tumor (203, 204). Endometrial cancer is classified into two types: type I - endometrioid endometrial cancer, amounting to 75% of cases, and type II - non-endometrioid, which can have clear-cell or serous histology. Endometrioid cancers more often show mutations in the Kirsten rat sarcoma viral oncogene homolog (KRAS) and Phosphatidylinositol 3-kinase catalytic subunit alpha (PI3KCA) and phosphatase and tensin homolog (PTEN) loss, while non-endometrioid cancers show human epidermal growth factor receptor 2 (HER-2) overexpression and mutations in p53 (205). Phosphoinositide-3-Kinase Regulatory Subunit 1 (PIK3R1) mutations are present in approximately 43% of endometrioid endometrial cancers and about 12% of non-endometrioid endometrial cancers, the mutated PI3KR1 leading to increased activation of the PI3K/AKT signaling pathway (206).

Oncogene expression and aggression factors in endometrial malignancies have been researched in comparison with normal endometrial tissue, and miRNA expression has been found to be significantly different. Moreover, among endometrial cancer types, such as papillary carcinoma and endometrioid carcinoma, miRNA patterns have been demonstrated to vary (207). miRNAs have the ability to link to the associated mRNA targets with or without perfect complementarity, with multiple target genes being simultaneously influenced by a single miRNA, either by means of the same or different cellular signaling pathways. Consequently, delivery of tumor suppressor miRNAs along with silencing of oncogenic miRNAs have been demonstrated to carry the potential to repair aberrations of the related signaling pathways in endometrial cancer (208, 209). For instance, in endometrial cancer, while miR-34b, which is linked to invasion and proliferation, can be overexpressed, along with miR-100, miR-99a, and miR-199b, the tumor-suppressive miR-34a is underexpressed (67, 210). Expressions of specific miRNAs in endometrial cancer cells have been found to be either elevated, such as miR-423, miR-210, miR-185, miR-7, or decreased, like miR-221, miR-let7e, miR-30c, when compared to normal endometrial tissue. To this extent, tumor-suppressor mRNAs have been found to be suppressed by the former miRNAs, thus promoting tumor cell growth, invasion, and metastasis. In turn, tumor suppressor miRNAs such as miR-221, miR-let7e, and miR-30c tend to inhibit oncogenic mRNAs, their lowered levels enabling carcinogenesis (211). Further thorough research conducted by Chung et al. has identified another miRNA cluster that appears to be dysregulated in endometrial cancer. Using low-density arrays, they analyzed the expression of 365 human miRNAs in normal and endometrioid endometrial cancer and identified a cluster of dysregulated miRNAs, including miR-7, miR-194, miR-449b, and miR-204. They also discovered that, by overexpressing miR-204, which is involved in the regulation of Forkhead box C1 (FOXC1), cell migration and the number of invasive cells could be inhibited (212).

In endometrial cancer cells, Dong et al. have identified 23 miRNAs as being dysregulated, mainly as a result of the mutated p53 (213). miR-130b among them, which is decreased in endometrial cancer relative to normal tissues, has the ability to target the key EMT promoter gene ZEB1 and to revert mutant p53-induced EMT/CSC of endometrial cancer cells (213, 214). Furthermore, miR-205 along with the miR-200 family inhibits EMT by regulating the E-cadherin dependent transcription of repressors ZEB1 and ZEB2 (75, 215). Overexpression of miR-200b in adenocarcinoma cells has also been found to inhibit the expression of the tissue inhibitor of metalloproteinase-2 (TIMP2), and increase the matrix metalloproteinase level (MMP), revealing the implication of miR-200b in endometrial cancer metastasis (216). EMT plays an important role in promoting chemoresistance and tumor cell invasion, with cancer biology studies and genetic evidence showing that the PI3K/AKT signaling pathway is the main mechanism controlling EMT features, despite its effects on cancer cell survival and proliferation (217). During EMT, adhesive and polarizing capabilities of the epithelial cells are lost, therefore gaining invasive and migratory behaviors which promote cancer invasion and metastatic spread. Judging by the suppressive action of miR-205 on EMT, some studies have suggested that high levels of miR-205 can be regarded as a marker of early-stage cancer, thus leading to an improved prognosis (218, 219).

Further on, the overexpression of miR-21 in endometrial cancer tissues and downregulation of PTEN through linking to 3’UTR of PTEN mRNA has been shown to promote cell proliferation (220). Similarly, decreased expression of PTEN associated with an increased expression of miR-205 in endometrial cancer has been linked to smaller overall patient survival rates, suggesting the link between miR-205 and the 3’-UTR of PTEN mRNA in endometrial cancer cells (84). Additional research has indicated that the transfection of endometrial cancer cells with miR-183 resulted in PTEN protein expression reduction. However, it is yet to be clear if miR-183 can suppress 3’-UTR of PTEN mRNA in endometrial cancer cells (84, 221).

Endometrial cancer proliferation has also been revealed to be promoted by the abnormal regulation of the Notch pathway (222). The Notch signaling cascade is initiated by the interactions between specific ligands and receptors, and Guo and colleagues have demonstrated that, by repressing Notch signaling, the tumor suppressor lncRNA human maternally expressed gene 3 (MEG3) inhibits cell proliferation in endometrial carcinoma (223). The initiation of the Notch signaling pathway is triggered by the ligand-receptor interaction, followed by the intramembranous proteolytic cleavage of the Notch receptors, which ensures the delivery of an active form of the Notch intracellular domain (NICD). NICD shows positive regulation of target genes such as the hes family bHLH transcription factor 1 (HES1), by acting as a transcriptional activator following nuclear translocation. HES1 has a particular effect on cell proliferation, by acting as a transcriptional repressor that negatively regulates genes such as the cyclin-dependent kinase inhibitor p27Kip1 (224). The Notch signaling pathway is also thought to be involved in the interaction between miR-184 and cell division cycle 25 A (CDC25A) protein. By examining the CDC25A mRNA sequence and identifying an optimal binding site for miR-184, Chen et al. have recently found that miR-184 overexpression significantly reduced CDC25A protein levels, thus hindering endometrial carcinoma cell growth and invasion. Conversely, they have also highlighted that downregulation of Notch receptors (NOTCH 1,2,3,4) and target gene HES1 induced by miR-184 can be overturned by the overexpression of CDC25A (225).

### miRNAs in Vulvar Cancer

Vaginal and vulvar carcinomas are rather rare diseases, adding up to 4% of gynecological cancers worldwide (226). According to the Centers for Disease Control and Prevention, approximately 5% of US females with genital tract malignancies are diagnosed with vulvar and vaginal neoplasm (227). The management of vulvar neoplasia is highly dependent on early diagnosis, clinical, and pathological degree of the tumoral process at the time of detection and on the emergence of loco-regional lymph node metastases (228). Therefore, the identification and detection of specific miRNAs for this type of cancer may help better address this public health concern and patients’ needs. Some types of miRNA molecules make up specific profiles for vulvar carcinoma, with some authors proposing them as biomarkers for both early diagnostics and therapeutic signaling targets in personalized treatments (229). However, so far only a few suitable references regarding microRNAs expressed in vulvar cancerous lesions, especially vulvar squamous cell carcinoma, have been reported (230).

There are two different pathways by which vulvar cancers develop. Squamous cells leading to vulvar squamous cell carcinomas (VSCC) amount to about 80% of all vulvar malignant tumors (230, 231). Most of these tumors, especially among young women, are linked to high-risk human papillomavirus (hrHPV) infections (231, 232) and are associated with other risk factors such as immunosuppression and smoking (233). Other types of carcinomas may appear in the context of chronic inflammatory skin diseases such as lichen sclerosus (LS) or may constitute differentiated vulvar intraepithelial malignancies, basal cell carcinomas, malignant melanomas, Paget disease or Bartholin gland carcinoma (232, 234). Genetic and epigenetic changes in vulvar lesions were reported in quite a few studies, where vulvar intraepithelial neoplasia (VIN) and VSCCs were correlated with HPV infection. Some of these studies have identified p53 as an altered signaling pathway, where NOTCH1 mutations were often detected (235, 236). Some studies have reported somatic mutations in higher range regarding HPV-negative tumors compared to HPV-positive tumors, where mutations of TP53 were detected (237). The number of genetic changes, however, also depends on the cancer stage, increasing the number of dysregulated signaling pathways and the altered signaling molecules, thus elevating the grade of dysplasia (235). Other studies reported different altered signaling processes related to VSCC. For instance, in VSCC it has been shown that miR−3147 regulates the Smad4 pathway by repressing mRNAs of Bax, Bim, p21, and PAI-1 genes, thus increasing both migration and invasion (86), while miR−590−5p promotes malignant cellular processes by upregulating the target gene TGFβRII (228). The extensive roles of miRNAs in vulvar carcinoma have also been recently investigated by Yang et al., who suggested that miR−4712−5p may promote carcinogenesis by targeting PTEN and could facilitate VSCC growth and invasion through the alteration of the PTEN/Akt/p−GSK3β/cyclin D1 signaling pathway (231).

Further on, the expression of miRNA molecules involved in vulvar carcinomas has also been explored by Yang and colleagues, so as to elucidate their mechanism of action in correlation with the expression levels of transforming growth factor-β (TGF-β) and Smad pathway factors (228). They found that 157 miRNA molecules were expressed in a significantly altered manner in this type of carcinoma. They concluded that while some miRNA molecules like miR-590-5p, miR-182-5p and miR-183-5p were upregulated, others were downregulated, especially miR-603, miR-103a-3p, and miR-107. Overexpression of miR-590-5p was found to induce decreased mRNA and protein levels of TGFβIIR, thus altering the TGF-β/Smad signaling pathway, and therefore facilitating the generation of malignant cellular changes along with sentinel lymph node metastasis (228). Further on, Zalewski et al. have recently conducted a study aimed at researching the expression levels of several miRNAs in plasma of patients harboring the same type of lesions: vulvar intraepithelial neoplasia and vulvar carcinoma. Six microRNAs (hsa-miR-425-5p, hsa-miR-191-5p, hsa-miR93-5p, hsa-miR-423-5p, hsa-miR-103a-3p and hsa-miR-16-5p) were analyzed. Of these, hsa-miR-93-5p and hsa-miR-425-5p were the most appropriate genes that could be used as internal controls for quantitative miRNA expression in these kind of rare gynecological malignancies (226).

## Resveratrol: The miRNA Controlling Compound

Inflammation is a non-specific immune response of the human body to a tissue injury, toxic compounds, damaged cells, irritant molecules or allergens. Inflammation is associated with both healing and destruction of the tissue from the surrounding area. In response to inflammation, the immune system coordinates a large variety of mediators. The hallmark of inflammation is the recruitment of the leukocytes in the peripheral zone of the tissue (238, 239). There are two pathways that connect inflammation with cancer: the intrinsic pathway, based on genetic alterations that induce neoplasia and inflammation, and the extrinsic pathway, which increases cancer risk by inflammatory conditions (240). Several miRNAs have been shown to play a part in both cancer and inflammation, with the most investigated being miR-21, miR-125b, and miR-155. For instance, miR-155, which is elevated in lymphomas and human leukemias, is known to be involved in erythropoiesis and myelopoiesis, B-cell maturation, Th1 differentiation, gene conversion, IgG1 production, B- and T-cell homeostasis and in the overall regulation of the immune response (91).

Found in grapes and berries, resveratrol is a natural polyphenolic antioxidant. Recent studies have shown that resveratrol has properties in cancer and cardiovascular prevention. It was first shown that resveratrol can inhibit tumor promotion and progression in skin cancer studied on mice (241, 242). Resveratrol 4-hydroxystyryl and m-hydroxyquinone moieties seem to be significant due to their inhibitory properties concerning various enzymes, such as cyclooxygenases and lipoxygenases that produce pro-inflammatory factors starting from arachidonic acid and protein kinases (243). Resveratrol is a pleiotropic element known to target a series of proteins in patients diagnosed with ovarian cancer, particularly HES1 and NOTCH2 in CAOV-3 and OVCAR-3 cells. Resveratrol has been shown to downregulate WNT2 in CAOV3 cells and the nuclear cumulation of B-catenin was reduced. Furthermore, resveratrol notably reduced OVCAR-3 cell nuclear cumulation of STAT3. Despite the evidence about resveratrol targeting multi-proteins, it is necessary to determine the particular features of the mechanism involved in the signaling pathways of this interwoven network (244).

The effect on NOTCH signaling of resveratrol is known to be context-dependent. Resveratrol has an inhibiting effect when NOTCH signaling is oncogenic. However, resveratrol is potentiating the tumor-suppressive action when NOTCH signaling has suppressive tumoral action. c-Myc (oncogenic transcription factor), an mRNA protein, was repressed by resveratrol in treated breast cancer cells. In consequence, c-Myc decrease resulted in the diminishing of miR-17 and pri-miR-17-92, whilst c-Myc overexpression significantly increased miR-17 and pri-miR-17-92 (245).

Studies have shown that this antioxidant also impedes the proliferation of MDA-MB-231-luc-D3H2LN breast cancer cells and the attention was directed to miRNA analysis, being revealed that resveratrol generates the expression of miR-141 and miR-200c within these cells (82, 246, 247). Besides, various genes and biological signaling pathways were regulated by resveratrol. One example is the p53 pathway which, once activated by resveratrol, leads to cell death with the implication of miRNAs. Several miRNAs like miR-34a, miR-424, and miR-503 can impede breast cancer development being downregulated by resveratrol through the p53 pathway, thus inhibiting HNRNPA1, whose expression is connected with cancer spread (248, 249). The effects of resveratrol on miRNAs invariably prove to be essential due to its anti-cancer, anti-inflammation, and anti-metastatic properties. miR-663, miR-155, and miR-21 are implicated in the regulation of native immunity, cell proliferation, tumor development, and metastasis apparition, which suggests that the capacity of resveratrol to behave as an anti-proliferation, anti-tumor, and anti-inflammatory agent at the same time arises from its ability to promote the expression of endogenous miRNAs, thus having the capacity to globally affect the cell proteome (91).

## Conclusions and Future Perspectives

Due to their high incidence and mortality rate worldwide, gynecological cancers have become a global public health problem. In this review article, while summarizing the ﬁndings in the literature regarding the roles of miRNAs in cancer-relevant processes, speciﬁcally in the context of gynecological cancers, we have also focused on implications of miRNA signaling pathways and their role in regulating oncogenic processes in breast, ovarian, cervical, vulvar and endometrial cancer.

miRNAs have arisen as signaling molecules in virtually all biological pathways in different forms of cancer, including gynecological neoplasia, and it has been proven that these molecules could regulate various processes involved in tumorigenesis. Considering the ability of a single miRNA to target hundreds of mRNAs, expressions of aberrant miRNA are liable for the deregulation of signaling pathways that control cancer-associated processes. Understanding the mechanism by which these changes within intercellular and intracellular signaling pathways occur represents a challenge for the survival of women who are detected with advanced and/or recurrent gynecological malignancies. As the important functions of miRNAs in gynecological cancer are being deciphered, their potential use as prognostic and/or diagnostic markers is evidenced by a long list of studies. Therapeutic strategies involving the reintroduction of lost miRNAs in cancer or inhibition of oncogenic miRNAs are steadily being developed. Various signaling pathways have been identified and described to be modified in gynecological cancers. The occurrence of oncogenic mutations may result in overexpression of the affected genes or in the production of mutated proteins whose activity is downregulated. Such proteins could be involved in signaling pathways that are implicated in many physiological cellular processes, like inflammatory cytokine production, proliferation, senescence and apoptosis, metastasis, and drug resistance

miRNAs such as miR-145, have been identified as central players in cervical carcinogenesis, whereas it has been demonstrated that miR-125b, miR-145, miR-21, and miR-155 have pivotal roles in breast neoplasia. miR-200 and let-7 have been described as key modulators in ovarian malignancies and miR-185, miR-210, miR-423, let-7c, miR-205, and miR-429 have been associated with oncogenesis, invasion, and metastasis in endometrial carcinoma. Resveratrol, on the other hand, has the capacity to behave as an anti-proliferation, anti-tumor, and anti-inflammatory agent at the same time, due to its expression on the endogenous miRNAs, having the capacity to globally affect the cell proteome.

In this review article, we have summarized new research ﬁndings regarding the importance that miRNAs have in cancer-relevant processes, speciﬁcally concerning the gynecological field, and about their significant role in the disruption of cancer-related signaling pathways, so as to improve the overall medical management of gynecological malignancies.

## Author Contributions

Conceptualization: FD, CC, CD, EB. Methodology: SC, JX, DC. Investigation: NS, MR. Resources, NS, D-VP. Writing—original draft preparation: FD, CC, CD, EB. Writing—review and editing: JX, XL, DC. Supervision: XL, SC, D-VP. Funding acquisition: NS, D-VP. All authors contributed to the article and approved the submitted version.

## Funding

This work was supported by grants of the Romanian Ministry of Research and Innovation, CCCDI-UEFISCDI, project number PN-III-P1-1.2-PCCDI- 2017-0833/68/2018 and PN-III-P1-1.2-PCCDI-2017-0820/67/2018 within PNCDI III.

## Conflict of Interest

The authors declare that the research was conducted in the absence of any commercial or financial relationships that could be construed as a potential conflict of interest.
